# Clinical Safety and Feasibility of Minimally Invasive Colectomy Under Neuraxial Anesthesia in Frail Patients: Preliminary Case Series

**DOI:** 10.3390/jcm14165822

**Published:** 2025-08-18

**Authors:** Filippo Carannante, Valentina Miacci, Renato Ricciardi, Giuseppe Pascarella, Vincenzo Schiavone, Massimiliano Carassiti, Gianluca Costa, Marco Caricato, Felice Eugenio Agrò, Gabriella Teresa Capolupo

**Affiliations:** 1 UOC Chirurgia Colorettale, Fondazione Policlinico Universitario Campus Bio-Medico di Roma, Via Àlvaro del Portillo 200, 00128 Rome, Italy; valentina.miacci@policlinicocampus.it (V.M.); g.costa@policlinicocampus.it (G.C.); m.caricato@policlinicocampus.it (M.C.); g.capolupo@policlinicocampus.it (G.T.C.); 2 Department of Anesthesia, Intensive Care and Pain Medicine, Fondazione Policlinico Universitario Campus Bio-Medico di Roma, Via Alvaro del Portillo, 00128 Rome, Italy; renato.ricciardi@policlinicocampus.it (R.R.); g.pascarella@policlinicocampus.it (G.P.); m.carassiti@policlinicocampus.it (M.C.); f.agro@policlinicocampus.it (F.E.A.); 3 Department of Advanced Biomedical Science, University of Naples Federico II, 80131 Naples, Italy; vincenzoschiavone92@gmail.com; 4 Department of Medicine and Surgery, Università Campus Bio-Medico di Roma, Via Àlvaro del Portillo 200, 00128 Rome, Italy; 5 General Surgery, Department of Life Sciences, Health and Health Professions, Link Campus University, 00165 Rome, Italy

**Keywords:** colorectal surgery, colon cancer, minimally invasive surgery, frail patient, neuroaxial anesthesia, locoregional anesthesia

## Abstract

**Background/Objectives:** General anesthesia is the most widely used anesthesia technique for major abdominal surgery, but it may have a longer recovery time, high cost, and environmental impact. In addition, general anesthesia may be contraindicated in some frail patients. Our study aims to evaluate the feasibility and safety of performing colorectal surgery with a minimally invasive technique in frail patients under spinal anesthesia. **Methods:** From June 2021 to January 2022, 39 consecutive frail patients, undergoing elective laparoscopic colorectal oncological resection surgery under neuraxial anesthesia at the Colorectal Surgery Unit of the Fondazione Policlinico Campus Bio-Medico in Rome, were selected. A retrospective analysis prospectively maintained database of these patients was performed. **Results:** In all 36 patients, the surgery was successfully completed under spinal anesthesia and laparoscopic technique. Some patients experienced mild abdominal pain between I and II POD (Post Operative Day) (Visual Analogue Scale between 3 and 5) and were treated with oral analgesics as needed. No patients experienced episodes of vomiting or nausea after surgery with gas channeling in I POD. The average hospital stay was about 4 days (range 3–7). No patient required ICU (Intensive Care Unit) admission, and 30-day mortality was 0. **Conclusions:** Our preliminary data show that performing major surgery with a minimally invasive technique under spinal anesthesia can be feasible and safe, if performed by experienced operators, and can be a viable alternative for the treatment of frail and/or high-risk patients.

## 1. Introduction

General anesthesia (GA) has long been considered the standard anesthetic technique for most abdominal surgeries due to its ability to provide profound sedation, complete muscle relaxation, and airway control. However, this approach is not without limitations. While GA enables the performance of complex surgical procedures, it is associated with a range of drawbacks, including adverse drug effects, longer hospitalization, increased postoperative complications, higher healthcare costs, and a growing environmental burden due to the release of volatile anesthetic agents into the atmosphere.

In contrast, regional anesthesia (RA), including spinal and epidural techniques, is more commonly used for minor or superficial procedures such as hernia repairs or abdominal wall surgeries. It is particularly indicated for patients with increased anesthetic risk, where avoiding GA may offer a safer alternative. Despite these benefits, the use of regional anesthesia in major abdominal surgery has remained limited, largely due to concerns about its adequacy for longer or more complex procedures and its perceived lack of versatility compared to GA [1].

In recent years, however, the surgical and anesthetic communities have shown renewed interest in the application of RA techniques, particularly in the context of minimally invasive surgery (MIS) and enhanced recovery protocols. This resurgence is motivated in part by the pressing need to reduce perioperative morbidity in frail and elderly patients, who have a diminished capacity to tolerate physiological stressors such as surgery and anesthesia, placing them at higher risk of adverse outcomes [2,3,4].

In parallel, the development and implementation of Enhanced Recovery After Surgery (ERAS) protocols [5,6] have contributed significantly to the resurgence of RA. Among its core components is the use of multimodal, opioid-sparing analgesia—an area where RA plays a central role. Although the standard application of RA within ERAS often involves its use in combination with general anesthesia (e.g., spinal or epidural analgesia as an adjunct to GA), there is increasing interest in using RA as the primary anesthetic technique, particularly when paired with minimally invasive surgical approaches. This combination may allow for optimal pain control, earlier mobilization, and fewer complications, without the drawbacks of general anesthesia.

Moreover, the environmental impact of general anesthesia has become an increasingly important consideration in modern clinical practice. Inhalational anesthetic agents such as desflurane and sevoflurane are potent greenhouse gases with high global warming potentials. The routine use of GA in operating theatres contributes to healthcare’s carbon footprint—a concern that is particularly relevant in the context of global efforts to reduce emissions [7].

Despite these advantages, the application of spinal anesthesia in major oncologic abdominal surgery remains largely underexplored in the current literature. Most published experiences focus on its use in small or medium-sized procedures, or in emergency settings where patients were deemed unfit for general anesthesia [8,9].

Given this background, our study aims to investigate the feasibility, safety, and postoperative complications, as primary outcomes, of using spinal anesthesia as the primary anesthetic technique in minimally invasive colorectal cancer surgery in frail or high-risk patients. By evaluating perioperative outcomes in a cohort of patients managed with this approach, we seek to contribute to the growing body of evidence supporting the use of regional anesthesia in major abdominal procedures.

## 2. Materials and Methods

This is a preliminary case series. From June 2021 to January 2022, 39 consecutive frail patients, undergoing elective laparoscopic colorectal oncological resection surgery under neuraxial anesthesia at the Colorectal Surgery Unit of the Fondazione Policlinico Campus Bio-Medico in Rome, were selected. A retrospective analysis of prospectively collected data was performed. The inclusion criteria were patients over 65 years old; elective oncological surgery of the colon or rectum performed with a laparoscopic approach and patients classified as frail using five tests: Activities of Daily Living (ADL), the Instrumental Activities of Daily Living (IADL), the Cumulative Illness Rating Scale (CIRS), the Physical Activity Scale for the Elderly (PASE) and the Mini Mental State Examination (MMSE). Preoperative risk was assessed with an anesthesiologist-assigned American Society of Anesthesiologists (ASA) score. All included patients underwent the anesthesiologic protocol described in the section “Anesthesiologic management” (see below). We excluded all patients under 65 years old, who refused to give informed consent, and patients who underwent emergency surgery, suffered from inflammatory diseases of the colon, or certified allergy to drugs used for neuraxial anesthesia and low or ultralow rectal resection to minimize the bias. The purpose of our study is to evaluate the feasibility and safety of performing minimally invasive oncological colorectal surgery under neuraxial anesthesia, with a focus on assessing short-term outcomes. We evaluate the admission to the ICU, postoperative complications (anastomotic leakage, ileus, bleeding, nausea/vomiting, pneumonia, urinary tract infection), postoperative pain, hospital stay, 30-day readmission, 30-day re-intervention, and 30-day mortality. Postoperative complications have been reported and categorized according to the Clavien–Dindo system. We performed a structured follow-up for all included patients (1-month, 6 months, and 1-year), and we have no patients lost to follow-up. This study was conducted according to the ethical principles outlined in the Declaration of Helsinki, and the approval of the Institutional Ethics Committee was obtained (303.24 CET2 cbm). The patients have been informed about the anesthesiological and surgical procedures, and informed consent has been obtained. This study adhered to the CARE checklist guidelines [10].

## 3. Anesthesiologic Management

All the patients underwent combined spinal-epidural anesthesia (CSE) at the T12-L1 intervertebral space after ultrasound evaluation of neuraxial structures. Ropivacaine 12 mg, fentanyl 20 mcg, and dexmedetomidine 5 mcg were diluted in 5 mL of saline solution and injected intrathecally. Intrathecal Fentanyl was used to shorten the onset of spinal anesthesia, while dexmedetomidine was administered to prolong neuraxial blockade. Subsequently, a peridural catheter was placed to manage postoperative analgesia according to ERAS recommendations. Before starting surgery, the extension of the sensory block to the T4 dermatome was verified through the pinprick test. During surgery, a mild sedation was administered through an i.v. target-controlled infusion remifentanil (0.8–1 ng/mL effect site concentration). Postoperatively, multimodal analgesia was given through i.v. dexamethasone 4 mg, acetaminophen 1 g every 8 h, ketorolac 30 mg every 12 h. In addition, a continuous infusion of ropivacaine 0.2%, 5 mL/h, was given through an epidural catheter during the first 48 h postoperatively. In all patients, an arterial line was placed for real-time blood pressure monitoring, and electrocardiography and pulse oximetry were continuously recorded throughout the procedure. Intraoperative hypotension (defined as a systolic blood pressure < 90 mmHg or >20% decrease from baseline) was treated with incremental doses of ephedrine (5–10 mg), with intravenous fluids administered as appropriate. Bradycardia with a heart rate < 50 bpm was managed with intravenous atropine 0.5 mg.

## 4. Statistical Analysis

Statistical analysis was carried out using StataCorp 2019 STATA Statistical Software: release 16 (College Station, TX, USA: StataCorp LLC) and R version 4.1.2 (1 November 2021), (R Foundation for Statistical Computing, Vienna, Austria) by two blinded authors (ACo/FCap). Descriptive dichotomous data and counts were presented in frequencies, whereas continuous data were presented as mean values ± standard deviations (SD) and/or median with 25–75 Interquartile Range (IQR) and minimum-maximum range.

## 5. Results

**Figure 1:**
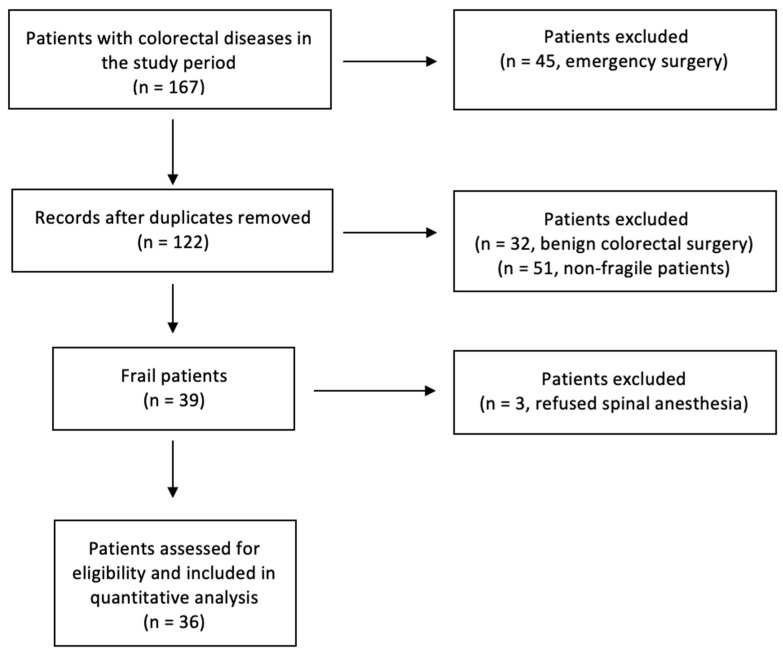
Flowchart diagram, following inclusion and exclusion criteria, which, starting from the whole number of patients treated at our institution in the study period, explains how we reached our sample size.

Thirty-nine patients, classified as frail, were enrolled in our analysis. Three of these patients refused surgery under spinal anesthesia, so 36 patients were included in the study (Figure 1). The average age was 80.9 years. The average operation time was 176 min. In all patients, the operation was successfully completed under neuraxial anesthesia and with a laparoscopic technique. Abdominal wall analgesia with the preoperative Tap Block technique was performed in all patients. The most common comorbidities were identified in all patients and are shown in Table 1.

29.4% of the patients suffered from COPD, 88.2% from hypertension, 35.3% from diabetes, and 47% from obesity.

All patients in the postoperative period followed the indications of the ERAS protocol for colorectal surgery: rehabilitation and early re-feeding and mobilization (same day of surgery or in I POD), no drain or nasogastric tube used, and removal of vesical catheter in I POD. All patients had a preadmission education and counselling with anesthesiologists, surgeons, stoma nurses, nutritionists, and hematologists. A total of 30 patients had first flatus in I POD, 4 patients in III POD, and 2 patients in IV POD. The hospital stay average was about 4.5 days (range 3–8).

According to the Clavien–Dindo score, 13 patients (35.3%) had complications. Grade I complications were encountered in 8 patients (22.2%), grade II complications in 2 patients (5.88%), and grade III complications in 2 patients (5.88%). In particular, 4 patients experienced episodes of vomiting or nausea after surgery, treated with drugs. Two patients (5.9%) had anastomotic leakage, treated with the use of an endosponge. One patient (2.8%) had endoluminal bleeding treated immediately by our endoscopist. One patient (2.8%) suffered from pneumonia treated with the use of IV antibiotics and oral antibiotics after discharge. No patients had wound infection. Four patients (11.8%) suffered from abdominal pain that required oral painkillers. No patients required admission to the intensive care unit, and no patients were readmitted to hospital after 30 days for complications. Re-intervention and the 30-day mortality was 0 (Table 2). We did a structured follow-up for all patients: 10 days; 30 days with abdominal CT scan, blood exams, and oncological appointment; 6 months with blood exams and abdominal ultrasound; 1 year with abdominal CT scan, blood exams, and a colonoscopy.

Pathological outcomes are reported in Table 3.

## 6. Discussion

General anesthesia (GA) remains the gold standard for intraoperative management in major abdominal surgery. Its widespread use is supported by its ability to ensure deep sedation, complete analgesia, and full muscular relaxation, which together provide optimal surgical conditions and airway control. However, this approach is not without drawbacks, particularly in the context of frail or comorbid patients. Complications associated with GA—ranging from postoperative pulmonary dysfunction to prolonged recovery and increased ICU admissions—have led to renewed interest in alternative anesthetic modalities [4,8,9].

Neuraxial anesthesia (NA), including spinal and epidural techniques, has traditionally been employed in superficial abdominal procedures and infra-umbilical surgery, such as urological, gynecological, and obstetric interventions. The use of spinal anesthesia in major abdominal surgery remains rare and typically limited to high-risk emergency cases where general anesthesia is contraindicated. In this context, our study aimed to evaluate the feasibility and safety of spinal anesthesia in the setting of elective oncologic colorectal surgery using a minimally invasive approach. This is particularly significant considering the Enhanced Recovery After Surgery (ERAS) [4,5] framework, which advocates for interventions that reduce surgical stress and promote faster postoperative recovery.

Historically, spinal anesthesia in abdominal surgery has been limited to emergency scenarios involving fragile patients with significant anesthetic risk. For instance, Hamad et al. [8] demonstrated in a feasibility study that laparoscopic cholecystectomy could be performed safely under spinal anesthesia in patients unfit for GA. Later, Kar et al. [9] reported a clinical series involving 291 patients, confirming the safety and reproducibility of spinal anesthesia in laparoscopic biliary surgery. However, these cases primarily involved relatively short procedures with minimal manipulation of abdominal organs.

The notion that spinal anesthesia could be used in more complex abdominal surgeries, such as colorectal resections, has received minimal attention. The scarcity of literature is not necessarily a reflection of ineffectiveness, but rather the result of entrenched reliance on GA and a lack of large-scale comparative studies. Our study contributes to filling this gap by presenting preliminary evidence supporting spinal anesthesia in elective colorectal cancer surgery.

One of the key advantages of neuraxial techniques is the avoidance of endotracheal intubation. GA requires the use of neuromuscular blocking agents and airway instrumentation to maintain oxygenation and prevent aspiration. These interventions, although standard, are associated with complications such as bronchospasm, airway trauma, postoperative pneumonia, and prolonged mechanical ventilation. In contrast, spinal anesthesia allows for the maintenance of spontaneous ventilation and eliminates the need for intubation and heavy sedation, significantly reducing pulmonary complications. In our cohort, we observed no cases requiring postoperative ventilatory support or ICU admission, an important finding in light of increasing ICU burden and patient frailty [11,12,13].

Additionally, neuraxial anesthesia reduces the pharmacological burden on patients by avoiding systemic administration of several classes of drugs. General anesthesia typically involves sedatives, opioids, neuromuscular blockers, and inhalational agents, each carrying specific risks. Of particular concern are opioids and neuromuscular blockers. Opioids may induce respiratory depression, ileus, nausea, vomiting, and oversedation—especially problematic in older adults or those with pre-existing respiratory compromise. Neuromuscular blockers, if not adequately reversed, can lead to residual paralysis, impaired diaphragmatic function, and hypoventilation.

By contrast, spinal anesthesia allows for high-quality analgesia using local anesthetics and, when necessary, low-dose intrathecal opioids, thereby minimizing systemic drug exposure. This targeted approach not only enhances pain control but also reduces the risk of postoperative delirium, cognitive impairment, and other medication-related complications [14,15,16].

The shift toward minimally invasive techniques and enhanced recovery protocols has created a more favorable landscape for the reintroduction of neuraxial anesthesia in major surgery. The ERAS program emphasizes early mobilization, minimal opioid use, and reduced perioperative stress—all goals well-aligned with spinal anesthesia.

Our findings reinforce the synergy between spinal anesthesia and ERAS. Patients in our series experienced effective intraoperative analgesia without the complications typically associated with general anesthesia. Additionally, the minimal use of opioids postoperatively aligns with ERAS goals of reducing opioid dependency and its associated side effects. This, in turn, facilitated earlier mobilization, improved bowel function, and reduced length of hospital stay—although these metrics were not the primary focus of our study and should be investigated further in future trials [17].

Notably, other surgical disciplines have long embraced neuraxial techniques as standard practice within their respective ERAS protocols. In obstetrics, combined spinal-epidural anesthesia is the gold standard for Caesarean sections, providing excellent analgesia while allowing patients to remain fully awake during surgery. In orthopedic surgery, spinal anesthesia has been linked with lower rates of deep vein thrombosis, pulmonary complications, and postoperative delirium compared to GA. The successes observed in these fields provide compelling evidence that similar benefits may be extended to colorectal surgery.

The physiological rationale for using spinal anesthesia in major abdominal procedures lies in its ability to block nociceptive transmission at the spinal cord level, attenuating the surgical stress response. This response, characterized by increased cortisol, catecholamines, and pro-inflammatory cytokines, contributes to immunosuppression, delayed healing, and higher rates of complications. By blunting this cascade, spinal anesthesia may confer a protective effect on immune function and reduce the incidence of surgical site infections and systemic complications [18].

Moreover, avoidance of mechanical ventilation and endotracheal intubation reduces barotrauma, atelectasis, and ventilator-associated pneumonia. These benefits are particularly significant in elderly patients or those with chronic pulmonary disease. In our study, no patient experienced major postoperative respiratory complications, underscoring the clinical relevance of these theoretical advantages [19].

Another benefit of spinal anesthesia is the potential for improved hemodynamic stability during surgery. While hypotension remains a known side effect, it is typically short-lived and manageable with vasopressors and fluid therapy. Importantly, spinal anesthesia avoids the cardiovascular depressant effects of inhalational agents and systemic opioids, which may be poorly tolerated in elderly or fragile patients [15,16,18].

Our case series offers a novel contribution to the existing anesthesia and surgical literature by addressing a significant gap in the application of neuraxial anesthesia for oncologic laparoscopic resections. While regional anesthesia has been increasingly explored in various surgical contexts, its use as the primary anesthetic technique in elective minimally invasive colorectal cancer surgery remains largely underreported. By focusing specifically on oncologic laparoscopic procedures, performed in frail and elderly patients, this study provides valuable data on the feasibility and safety of avoiding general anesthesia in a high-risk population.

A distinguishing feature of our study is the systematic incorporation of frailty screening into perioperative planning. Frailty is a critical yet often underappreciated predictor of postoperative outcomes, and its integration into anesthetic decision-making represents an important step toward personalized, risk-adapted care. By identifying patients with increased vulnerability, the study tailors anesthetic strategy to individual physiological resilience, aiming to improve outcomes and reduce complications.

Furthermore, the study is situated within an Enhanced Recovery After Surgery (ERAS) framework, aligning its anesthetic approach with contemporary best practices in perioperative care. The use of neuraxial anesthesia complements key ERAS principles, including multimodal analgesia, early mobilization, and opioid-sparing strategies, which collectively support faster recovery and reduced postoperative morbidity.

Together, these elements—focus on oncologic laparoscopic resections, integration of frailty assessment, and alignment with ERAS—underscore the study’s contribution to advancing sustainable, patient-centered anesthetic practices and expanding the role of regional anesthesia in complex surgical care.

Despite the encouraging results, several limitations must be acknowledged. The most significant is the small sample size, which limits the statistical power of our observations and the ability to generalize findings. Additionally, the retrospective nature of the analysis introduces potential biases, such as selection bias and reporting bias.

Our patient cohort was carefully selected, excluding those with contraindications to spinal anesthesia or significant anatomical complexities. Therefore, while our results are promising, they may not be directly applicable to all patient populations. Furthermore, long-term outcomes such as cancer recurrence, functional recovery, and quality of life were not assessed and should be included in future studies.

Another area for exploration is the optimal technique for spinal anesthesia in colorectal surgery. Questions remain regarding the best choice of anesthetic agents, dosage, and whether adjunctive sedation or peripheral nerve blocks should be routinely employed. Additionally, defining standardized criteria for patient selection and procedural protocols will be essential for broader adoption.

Future research should include prospective, randomized controlled trials comparing spinal and general anesthesia in colorectal surgery, with endpoints including postoperative morbidity, length of stay, patient satisfaction, and oncologic outcomes. Incorporating patient-reported outcomes would also provide valuable insight into the subjective experience and recovery trajectory of those undergoing spinal versus general anesthesia.

## 7. Conclusions

Our experience suggests that spinal anesthesia is a feasible and safe procedure in selected patients undergoing minimally invasive oncologic colorectal surgery. It aligns closely with ERAS principles, offering the potential for enhanced postoperative recovery, reduced opioid use, fewer respiratory complications, and lower ICU admission rates.

While general anesthesia will likely remain the preferred modality for complex and prolonged procedures requiring complete neuromuscular relaxation and airway control, spinal anesthesia offers a compelling alternative in cases where minimizing systemic burden and maximizing functional recovery are priorities.

In conclusion, this case series adds to the growing body of evidence supporting the expanded role of neuraxial anesthesia in abdominal surgery. It encourages a re-evaluation of established norms and paves the way for future research that may ultimately redefine anesthetic management in colorectal surgery. With careful patient selection, appropriate perioperative support, and integration into ERAS pathways, spinal anesthesia may offer significant clinical benefits and contribute to a paradigm shift in the care of surgical oncology patients.

## Figures and Tables

**Table 1:** Baseline Characteristics of the Study Population.; BMI: Body Mass Index.

	Spinal A.
** N. of patients**	39
** Refused spinal anesthesia**	3
** N. of patients studied**	36
** Age (year)**	80.9 ± 7.5
** Male gender (%)**	24 (64.7)
** BMI**	26.3 ± 3.8
** Associated medical conditions (%)**	
** COPD**	11 (29.4)
** Hypertension**	32 (88.2)
** Diabetes mellitus**	13 (35.3)
** Obesity**	17 (47)
** Tumor location (%)**	
** Right colon**	22 (58.8)
** Transverse colon**	2 (5.9)
** Left colon**	8 (23.5)
** Rectum**	4 (11.8)

**Table 2:** Intra and postoperative characteristics.

	Spinal A.
**N. of patients**	36
**Blood loss (range)**	150 mL (100–400)
**Conversion to open surgery (%)**	0 (0)
**ICU recovery (%)**	0 (0)
**Hospital stay (range)**	4.4 days (3–8)
**Operative time**	176 min
**Postoperative complications (%)**	
** Anastomotic leakage**	2 (5.9)
** Ileus**	5 (13.9)
** Bleeding**	1 (2.8)
** Nausea/vomiting**	4 (11.8)
** Pneumonia**	1 (2.8)
** Urinary tract infection (UTI)**	0 (0)
** Wound infection**	0 (0)
** Abdominal pain (NRS > 5)**	4 (11.8)
**Readmission**	0 (0)
**Reoperation**	0 (0)
**30-day mortality**	0 (0)

**Table 3:** Pathological reports.

**N. of Patients**	36
**T (tumor) (%)**	
T0	0 (0)
T1	0 (0)
T2	8 (23.5)
T3	24 (64.7)
T4	4 (11.8)
**N (nodes) (%)**	
N0	22 (58.8)
N1	10 (29.4)
N1a	4 (11.8)
N1b	6 (17.6)
N2	4 (11.8)
**M (metastasis) (%)**	
M0	34 (94.1)
M1	2 (5.9)

## Data Availability

The data presented in this study are available on request from the corresponding author.
